# Quasi-Static Optical Coherence Elastography to Characterize Human Corneal Biomechanical Properties

**DOI:** 10.1167/iovs.61.6.29

**Published:** 2020-06-15

**Authors:** Sabine Kling, Emilio A. Torres-Netto, Bogdan Spiru, Walter Sekundo, Farhad Hafezi

**Affiliations:** 1 OPTIC-team, Computer Vision Laboratory, ETH Zurich, Zurich, Switzerland; 2 Laboratory of Ocular Cell Biology, CABMM, University of Zurich, Zurich, Switzerland; 3 Department of Ophthalmology and Visual Sciences, Federal University of Sao Paulo, Sao Paulo, Brazil; 4 Department of Ophthalmology, Philipps University of Marburg, Marburg, Germany; 5 ELZA Institute, Dietikon/Zurich, Switzerland; 6 Medical Faculty, University of Geneva, Geneva, Switzerland; 7 USC Roski Eye Institute, Los Angeles, California, United States

**Keywords:** corneal biomechanics, strain, elastography, functional imaging, Bowman's membrane

## Abstract

**Purpose:**

Quasi-static optical coherence elastography (OCE) is an emerging technology to investigate corneal biomechanical behavior in situations similar to physiological stress conditions. Herein OCE was applied to evaluate previously inaccessible biomechanical characteristics of human corneal tissue and to study the role of Bowman's layer in corneal biomechanics.

**Methods:**

Human corneal donor buttons (*n* = 23) were obtained and Descemet's membrane and endothelium were removed. In 11 corneas, Bowman's layer was ablated by a 20 µm stromal excimer laser ablation. Buttons were mounted on an artificial anterior chamber and subjected to a pressure modulation from 10 to 30 mm Hg, and back to 10 mm Hg, in steps of 1 mm Hg. At each step, a spectral-domain optical coherence tomography scan was obtained. Displacements were analyzed by optical flow tracking, and strain over the entire stromal depth was retrieved from the phase gradient of the complex interference signal.

**Results:**

During pressure increase, corneal tissue moved upward (486–585 nm/mm Hg) but did not fully recover (Δ= 2.63 to 8.64 µm) after pressure decrease. Vertical corneal strain distribution was negative in the anterior and positive in the posterior cornea, indicating simultaneous corneal compression and expansion, respectively. Bowman's layer caused minor localized differences in corneal strain distribution.

**Conclusions:**

Corneal strain distribution is more complex than previously assumed, with a fundamental difference in mechanical response between the anterior and posterior stroma. Clinically, OCE technology might be used to monitor the progression of corneal ectatic diseases and to determine the success of corneal cross-linking.

The IOP is the major mechanical load imposed on and carried by ocular tissues. It undergoes physiological diurnal fluctuations^1^ of approximately 5 mm Hg, which induces radial expansion/contraction and accordingly corneal elongation/compression, that is, strain.^2^^,^^3^ Optical coherence elastography (OCE) is an emerging imaging technology based on subpixel displacement tracking to record such corneal strain maps with high-spatial resolution.^3^ Tissue strain is a property that is inherently related to its elastic modulus, which in turn determines corneal shape, and hence refractive power. Assuming a homogenous stress distribution across the corneal tissue, strain maps represent a direct measure of the elastic modulus. The assumption of homogenous stress distribution holds true in a thin-walled pressure vessel, that is, with a ratio of radius: thickness of factor 10 or higher. The human corneal dimensions—with a radius of approximately 7.7 mm and a thickness ^4^ of approximately 427 to 620 µm—correspond to a factor of 12 to 18. Given that the requirement of a thin-walled pressure vessel is met, and thus a homogenous stress distribution is expected from IOP, low-amplitude IOP modulation is a promising tool for biomechanical characterization close to its physiological stress environment and particularly well suited to resolve localized differences in corneal strain.

Previously, quasi-static biomechanical properties of human corneas have been investigated by means of uniaxial stress-strain extensometry,^5^^–^^7^ transverse biaxial extensometry,^8^ two-dimensional extensometry,^9^^,^^10^ whole eye inflation,^11^^–^^14^ Brillouin microscopy,^15^ and most recently OCE during anterior corneal compression.^16^ Each of these techniques shows limitations: ex vivo extensometry tests are destructive and apply loading conditions far from physiological conditions, whereas inflation tests are limited to corneal surface,^17^ apex displacement,^18^ and curvature changes.^19^ Brillouin microscopy is currently the most advanced method for in vivo spatially resolved corneal biomechanical evaluation.^20^ Brillouin scattering is a nonlinear optical effect leading to a frequency shift that correlates with material stiffness. However, the shift is not directly related to stiffness and depends on further parameters, such as mass density and refractive index, making it susceptible to imaging artifacts, for example, introduces dependency on hydration levels.^21^^,^^22^ Also, its slow imaging speed (12 seconds for a 40-pixel axial scan,^23^ compared with 15 µs for a typical 1024-pixel axial optical coherence tomography [OCT] scan) makes it susceptible to motion artifacts and prevents dynamic stiffness interpretation.

Recently, OCE was applied to determine corneal displacement during anterior corneal compression (flattening).^16^ However, displacement measures depend on further parameters, including corneal thickness and IOP and are less indicative of mechanical stiffness than strain distribution. Apart from quasi-static OCE approaches to assess corneal biomechanics, dynamic measures have been suggested, such as shear-wave velocity,^24^^,^^25^ and air-puff deformation analyses.^26^ A limitation of these techniques is that dynamic biomechanical characterization strongly depends on boundary conditions and geometric factors, such as mechanical fixation, IOP, and corneal thickness.

The purpose of the current study was to assess spatially highly resolved corneal strain distribution in human donor corneas during low-pressure inflation, and thus with loading conditions close to the physiological stress situation.

In addition, a particular interest was to investigate the biomechanical relevance of Bowman's layer. Histological and ultrastructural studies on ectatic human corneas after refractive surgery indicate slippage of collagen lamellae and fibrils in the residual stromal bed.^27^ It has been speculated that in the intact cornea, the densely packed collagen fibrils of Bowman's layer ensure maintenance of corneal shape. In line with this, atomic force measurements demonstrated that the elastic modulus of Bowman's layer is 3.3 times higher than that of the anterior stroma.^28^ However, atomic force measurements are susceptible to surface texture, and observed differences might have arisen from the removal of Bowman's layer by the excimer laser. Furthermore, in refractive surgery, the thickness of residual stromal bed^29^ and the presence of forme fruste keratoconus^30^ are considered more important for developing keratectasia than the ablation of Bowman's layer. When examining the stability of the full-thickness cornea in the presence and absence of Bowman's layer, no significant differences were found in tensile extensometry measurements.^31^ Similarly, when characterizing tensile material properties of 100-µm thin corneal flaps, no difference was found between flaps with and without Bowman's layer.^32^ Combining high-resolution OCE imaging with eye inflation, the impact of Bowman's membrane can be studied not only in terms of strain distribution, and hence biomechanical stiffness, but also in terms of displacement and curvature changes.

## Methods

### Ex Vivo Eye Specimen

A total of 23 human corneal buttons were obtained from the Philipps University of Marburg after removal of the Descemet membrane for DMEK (Descemet Membrane Endothelial Keratoplasty) in specimens with a consent for both transplantation and research. Corneal buttons were preserved in Optisol GS medium (Bausch & Lomb, Rochester, NY, USA) and kept refrigerated for a maximum of 14 days before measurement. Prior to measurements, corneal buttons were mounted on an artificial anterior chamber (Schwind eye-tech-solutions, Kleinostheim, Germany) and the partially degraded epithelial layer was removed to ensure a smooth surface and facilitate later surface detection. Subsequently, one group of corneal buttons (*n* = 11) underwent 20 µm excimer laser ablation (AMARIS 750; Schwind eye-tech solutions) within an optical zone of 10 mm diameter from the anterior stroma to remove Bowman's layer. In the other group (*n* = 12) Bowman's layer was left intact.

### Experimental Set-Up

A laboratory-built spectral-domain OCT system with a central wavelength of 878 nm and a bandwidth of 65 nm was used for the measurements. A single OCT volume scan (C-scan) contained 50 cross-sectional scans (B-scans), with each containing 1000 axial scans (A-scans). The lateral dimensions of the scanned area was 14 × 14 mm, covering the entire corneal tissue. The system has an axial resolution of 5.44 µm in air (approximately 3.96 µm in corneal tissue), and a lateral resolution (spot size) of 12.5 µm. Corneal buttons were mounted on an artificial anterior chamber (Barron Precision Instruments, Grand Blanc, MI, USA) for measurement. One of the two perfusion tubes was closed, the other one was connected to a closed-loop pressure control unit consisting of a 10-mL syringe mounted on a rigid stage, a stepper motor (Can Stack Linear Actuator 35DBM- L; Portescap SA, La Chaux-de-Fonds, Switzerland), a pressure sensor (NPC-100; Amphenol Advanced Sensors, Pforzheim, Germany), and a water column for buffering. Five minutes prior to starting the measurement, the intrachamber pressure was adjusted to 10 mm Hg. During the measurement cycle, pressure was first increased from 10 to 30 mm Hg in steps of 1 mm Hg, and then decreased by the same amount and variation. Each pressure step was kept constant for 12 seconds before an OCT C-scan was recorded to ensure that the induced deformation had reached a steady state. We conducted two different sets of measurements: the first set (*n* = 6 corneas) consisted of a full volumetric measurement and aimed at exploring the distribution of corneal strain. Here the measurement duration for one cornea was 13 minutes. The second set (*n* = 17 corneas) aimed at confirming the repeatability of the method. Due to memory limitations, the second set was restricted to cross-sectional measurements near the corneal apex. Here the measurement duration for a single cornea was 4 minutes.

### Displacement Tracking and Strain Computation

Axial strain was retrieved using a vector method for strain estimation in phase-sensitive OCT elastography,^33^ similar to as described recently.^3^ Briefly, phase differences ∠C_*IOP*_ were computed within a window size of 5 x 5 pixels according to:

$$\begin{array}{ccc} C_{\mathrm{IOP}}\left(z,x\right) & = & \sum_{j=-w_{z}}^{w_{z}}\sum_{k=-w_{x}}^{w_{x}}\left|A{\left(z+j,x+k\right)}_{\mathrm{iop}}\right|\\ & & \cdot \left|A{\left(z+j,x+k\right)}_{\mathrm{iop}+\Delta }\right|\\ & & \cdot \exp(-i\cdot ∠({A_{\mathrm{iop}}}^{*}\left(z+j,x+k\right)\\ & & \cdot A_{\mathrm{iop}+\Delta }\left(z+j,x+k\right)))\;, \end{array}$$

where *w*_z_ = 2 and *w*_x_ = 2 pixels, and *A* is the complex OCT signal. Subsequently, the phase gradient, which represents axial (vertical) strain ε_z_, was determined within a window size of 3 x 3 pixels by:

$$\begin{array}{ccc} {ɛ}_{z}\left(z,x\right) & = & \frac{\lambda_{\mathrm{mean}}}{4\pi \cdot asu}\cdot \frac{\partial ∠C_{\mathrm{IOP}}\left(z,x\right)}{\partial z}\\ & = & \frac{\lambda_{\mathrm{mean}}}{4\pi \cdot asu}\cdot ∠\sum_{j=-w_{z^{'}}}^{w_{z^{'}}}\sum_{i=-w_{x^{'}}}^{w_{x^{'}}}C_{\mathrm{IOP}}\left(z,x\right)*\\ & & {C_{\mathrm{IOP}}}^{*}\left(z+1+j,x+k\right), \end{array}$$

where *w*_z’_ = 1, *w*_x’_ = 1 pixel, *λ*_mean_ = 878 nm, and *asu* = axial sampling unit (3.26 µm). The corresponding resolution of strain images thus is 26 µm axially, and 112 µm laterally. To estimate the elastic modulus *E*, stress σ within a thin-walled pressure vessel was computed by:

$$\sigma =\frac{P\cdot R}{2\cdot th},$$

where *P* indicates the internal pressure, *R* the radius of curvature, and *th* corneal thickness. *E* is defined as the gradient in the stress-strain diagram and was computed accordingly:

$$E=\left|\frac{\Delta \sigma }{\Delta ɛ}\right|.$$

Axial (vertical) Δz and lateral Δx displacements were determined with the Lucas-Kanade method^34^ based on optical flow estimation:

$$\left[\begin{array}{cc} \frac{\partial {\left|C_{\mathrm{IOP}}\left(z,x\right)\right|}_{1}}{\partial x} & \frac{\partial {\left|C_{\mathrm{IOP}}\left(z,x\right)\right|}_{1}}{\partial z}\\ \vdots & \vdots \\ \frac{\partial {\left|C_{\mathrm{IOP}}\left(z,x\right)\right|}_{n}}{\partial x} & \frac{\partial {\left|C_{\mathrm{IOP}}\left(z,x\right)\right|}_{n}}{\partial z} \end{array}\right]\cdot \left[\begin{array}{c} \Delta x\\ \Delta z \end{array}\right]=\left[\begin{array}{c} \frac{\partial {\left|C_{\mathrm{IOP}}\left(z,x\right)\right|}_{1}}{\partial t}\\ \vdots \\ \frac{\partial {\left|C_{\mathrm{IOP}}\left(z,x\right)\right|}_{n}}{\partial t} \end{array}\right],$$

where |C_IOP_(*z*,*x*)| represents the intensity of the OCT signal, and *n* is the number of pixels within the applied window of 5 x 5 pixels. The $\frac{\partial }{\partial x}$ indicates the lateral gradient, $\frac{\partial }{\partial z}$ the axial gradient, and $\frac{\partial }{\partial t}$ the temporal gradient, that is, the difference between the deformed and reference image. The corresponding resolution of displacement images is 16 µm axially, and 70 µm laterally.

### Corneal Surface Detection and Curvature Analysis

**Figure 1.**
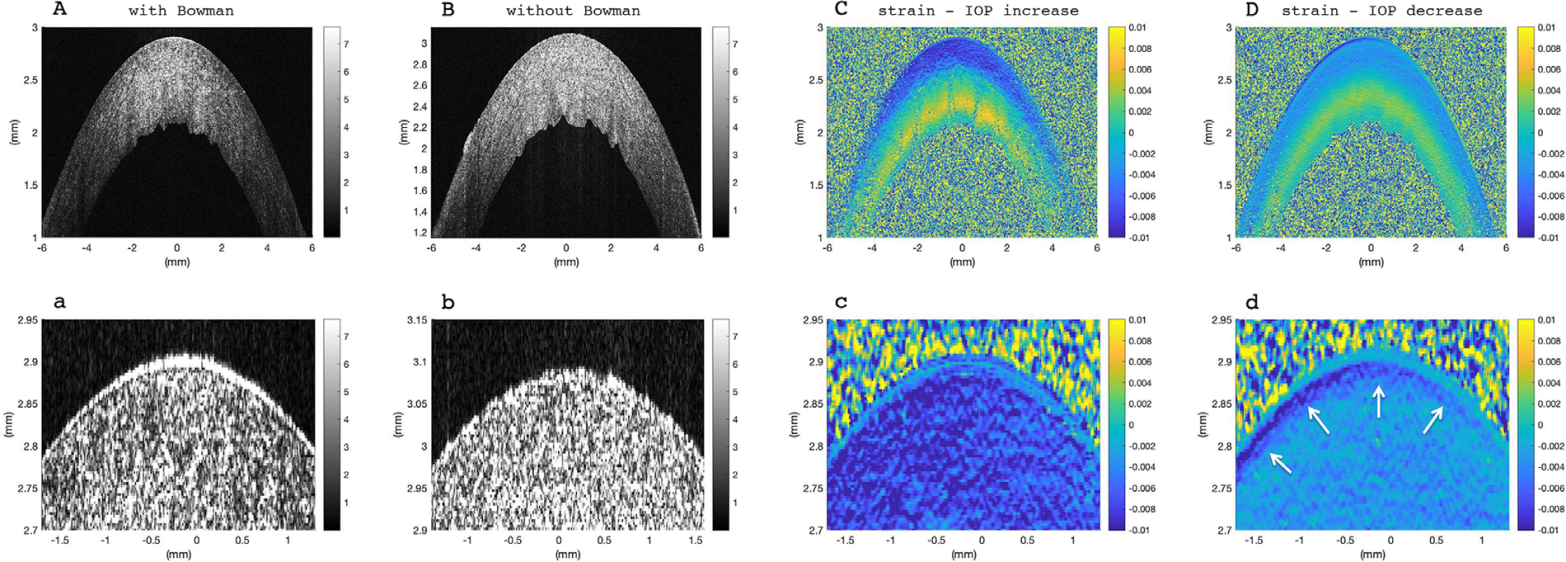
Corneal tissue at normal IOP (17 mm Hg). Morphological image: (**A**) with Bowman's layer, (**B**) without Bowman's layer, removed by excimer laser ablation. Strain distribution with Bowman's layer: (**C**) resulting from pressure increase by 1 mm Hg, (**D**) resulting from pressure decrease by 1 mm Hg. Panels (**a–d**) show a zoomed-in view of the anterior cornea from the image above. *White arrows* indicate an anterior structure that behaves different than the remaining stroma, particularly during pressure decrease. Corneas with and without Bowman's layer were indistinguishable from strain images.

Anterior corneal surface was detected with an image gradient-based processing algorithm. Surface detection was required for two purposes: (1) to flatten corneal tissue and compute the mean strain distribution across an optical zone of 5.4 mm diameter, and (2) to determine corneal curvature within an optical zone of 8 mm diameter. Corneal curvature was analyzed by computing the best fitting sphere, by means of a least-squares optimization. The posterior surface could not be detected due to severe structural irregularity resulting from the removal of the endothelial layer (see also Figs. 1A, 1D).

### Statistical Analyses

A two-tailed Student's *t*-test for independent samples was applied to test for differences between the deformation response in corneas with and without Bowman's layer. For comparisons between IOP increase and decrease within the same sample set, a two-tailed Student's *t*-test for paired samples was applied. A *P* value of <0.05 was interpreted as indicating significant differences.

## Results

Bowman's layer was visible in the morphologic OCT images (Fig. 1a), but was not detectable in phase, strain, or displacement maps. Some corneas presented an anterior region (white arrows Fig. 1d) that behaved slightly different than the rest of the anterior stroma, however, this was not limited to corneas with Bowman's layer. In all eyes, corneal strain maps were bilayered indicating negative, that is, compressive strains in the anterior stroma, and positive, that is, tensile strains in the posterior stroma—interestingly, both during IOP increase and decrease (Figs. 1C, 1D).

**Figure 2.**
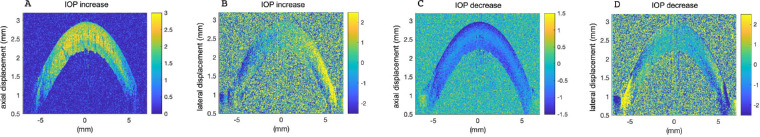
Mean corneal displacement at normal IOP in an intact cornea. (**A**) Vertical displacement and (**B**) lateral displacement in response to 1 mm Hg pressure increase. (**C**) Vertical displacement and (**D**) lateral displacement in response to 1 mm Hg pressure decrease. Mean corneal displacement was determined within a pressure range of 15 to 20 mm Hg to improve signal quality. Color unit is µm. A similar displacement was observed in the presence and absence of Bowman's layer.

Corneal displacement (Fig. 2) indicated radial expansion on pressure increase (Figs. 2A, 2B) and recovery on pressure decrease (Figs. 2C, 2D). Lateral corneal displacements were best recognizable in the corneal periphery (Figs. 2B, 2D), where the largest displacements are expected. Similar to strain maps, a bilayered deformation pattern was observed. The posterior cornea moved inward, whereas the anterior cornea moved outward. Note that the zero location of lateral displacements is located at the corneal apex. Similar deformation patterns were observed during IOP increase and decrease.

**Figure 3.**
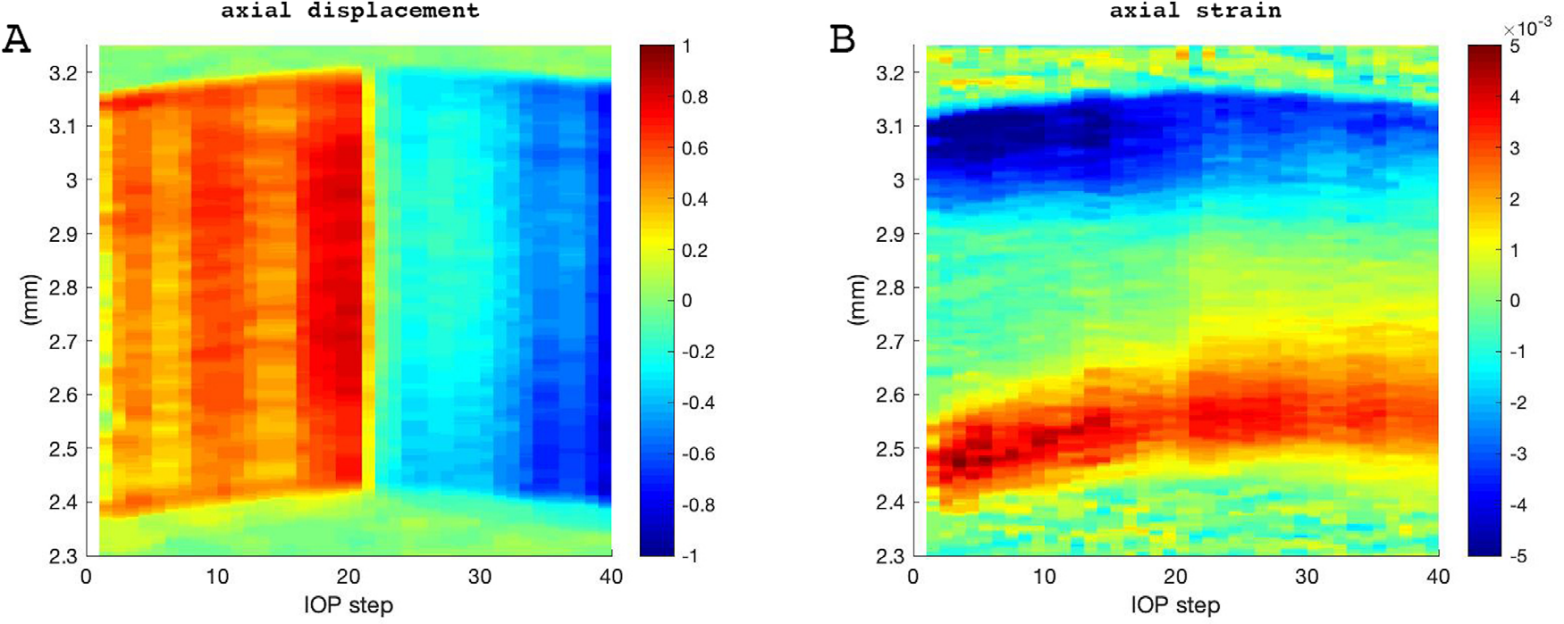
Corneal response of an intact cornea during pressure modulation from 10 to 30 mm Hg and subsequent decrease. Each vertical line represents the induced deformation averaged across an optical zone of 5.4 mm diameter in response to a 1 mm Hg pressure change. (**A**) Vertical displacement; colors indicate displacement in µm; (**B**) Axial strain map in vertical direction; colors indicate strain dimensionless. Similar displacement and strain evolution are observed in the presence and absence of Bowman's layer. Vertical displacement shows an immediate response to pressure decrease (pressure step 20). In contrast, corneal strain continues to increase even after pressure decrease.

Figure 3 presents a representative map of vertical displacement (Fig. 3A) and axial corneal strain (Fig. 3B) across corneal thickness as a function of IOP modulation. Vertical displacement reflects corneal apex displacement and clearly changed its sign on IOP decrease. In contrast, the induced corneal strain during IOP increase seemed to further progress even during IOP decrease, yet at smaller amplitude. Given the surprising strain behavior during IOP decrease, we validated the OCT system's ability to correctly retrieve axial strain during tension and compression. Supplementary Figure S1 demonstrates the deformation response of a purely isotropic material (polydimethylsiloxane), both under tension and compression. As expected, the sign of the strain changed when switching from tension to compression. Hence the inability to recover strain during IOP decrease seems to be a particularity of ex vivo corneal tissue.

**Figure 4.**
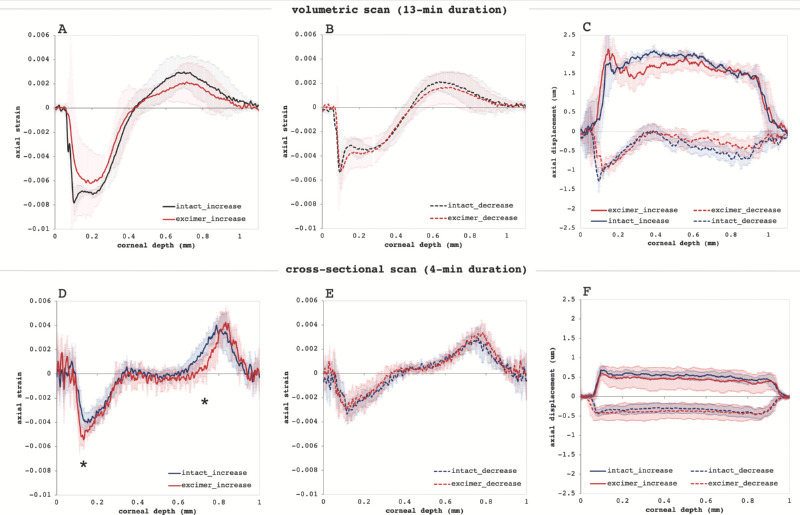
Deformation profile in response to a pressure increase/decrease by 1 mm Hg. Axial strain profile (**A,D**) during pressure increase, (**B,E**) during pressure reduction. The same profile, but smaller amplitudes were observed during pressure decrease (*p<0.05). (**C,F**) Vertical displacement profile. Larger displacements in response to pressure increase than decrease were observed.

Figure 4 shows the deformation profile across corneal thickness in terms of axial (vertical) apex displacement and axial strain. The strain profile (A) visualizes corneal strain distribution from the anterior (at 0 µm corneal depth) to the posterior surface. Largest negative strains ε_z_ occurred in the anterior cornea during IOP increase, −7.2 ± 0.4 ‰ (set 1) and −4.8 ± 0.6 ‰ (set 2) with Bowman, and −6.2 ± 1.7 ‰ (set 1) and −6.0 ± 0.8 ‰ (set 2) without Bowman. With shorter loading (set 2), anterior strain amplitude in corneas with Bowman's layer was significantly (*P* = 0.007) smaller than in corneas without. On IOP decrease, a reduction of anterior corneal strain amplitude was observed, both in samples with Bowman's layer (−4.3 ± 0.4 ‰ in set 1, *P* = 0.012; −4.3 ± 1.1 ‰ in set 2, *P* = 0.201) and without (−4.7 ± 1.7 ‰ in set 1, *P* = 0.038; −4.2 ± 0.7 ‰ in set 1, *P* = 0.006).

Largest positive strains occurred in the posterior cornea during IOP increase: maximal strain was 4.1 ± 0.3 ‰ (set 1) and 5.0 ± 0.7 ‰ (set 2) with Bowman, and 2.6 ± 1.7 ‰ (set 1) and 5.3 ± 0.9 ‰ (set 2) without Bowman. On IOP decrease, a reduction of posterior corneal strain amplitude was observed, both in samples with Bowman's layer (2.9 ± 0.4 ‰ in set 1, *P* = 0.107; 3.7 ± 0.7 ‰ in set 2, *P* < 0.001) and without (2.14 ± 1.5 ‰ in set 1, *P* = 0.035; 4.2 ± 0.8 ‰ in set 1, *P* = 0.051).

At certain corneal depth regions (indicated by asterisks in Fig. 4D) a significant (*P* < 0.05) difference between samples with and without Bowman's layer was observed with faster loading (set 2). Hence instead of a localized effect, Bowman's layer appears to have a more global mechanical effect reducing peak strains across the entire cornea.

The displacement profile (Figs. 4C, 4F) visualizes the corresponding absolute corneal movement. By comparing the displacement amplitude during the pressure increase and decrease cycle, it is evident that axial displacement is larger in response to pressure increase and does not fully recover. The displacement amplitude was substantially larger with slow compared with fast loading. Also, with slower loading (set 1), during pressure increase the midstroma showed a trend toward larger displacement than the above and below stromal layer (Fig. 4C); during pressure decrease, it did not get displaced at all, even though the above and below layer presented downward movement. The particular behavior of the midstroma at slow loading also becomes evident in Supplementary Video S1, which presents a time-lapse of the morphologic OCT image during the entire IOP modulation cycle. Interestingly, with faster loading (set 2), the stroma showed a more homogenous displacement profile (Fig. 4F), both during IOP increase and decrease.

**Figure 5.**
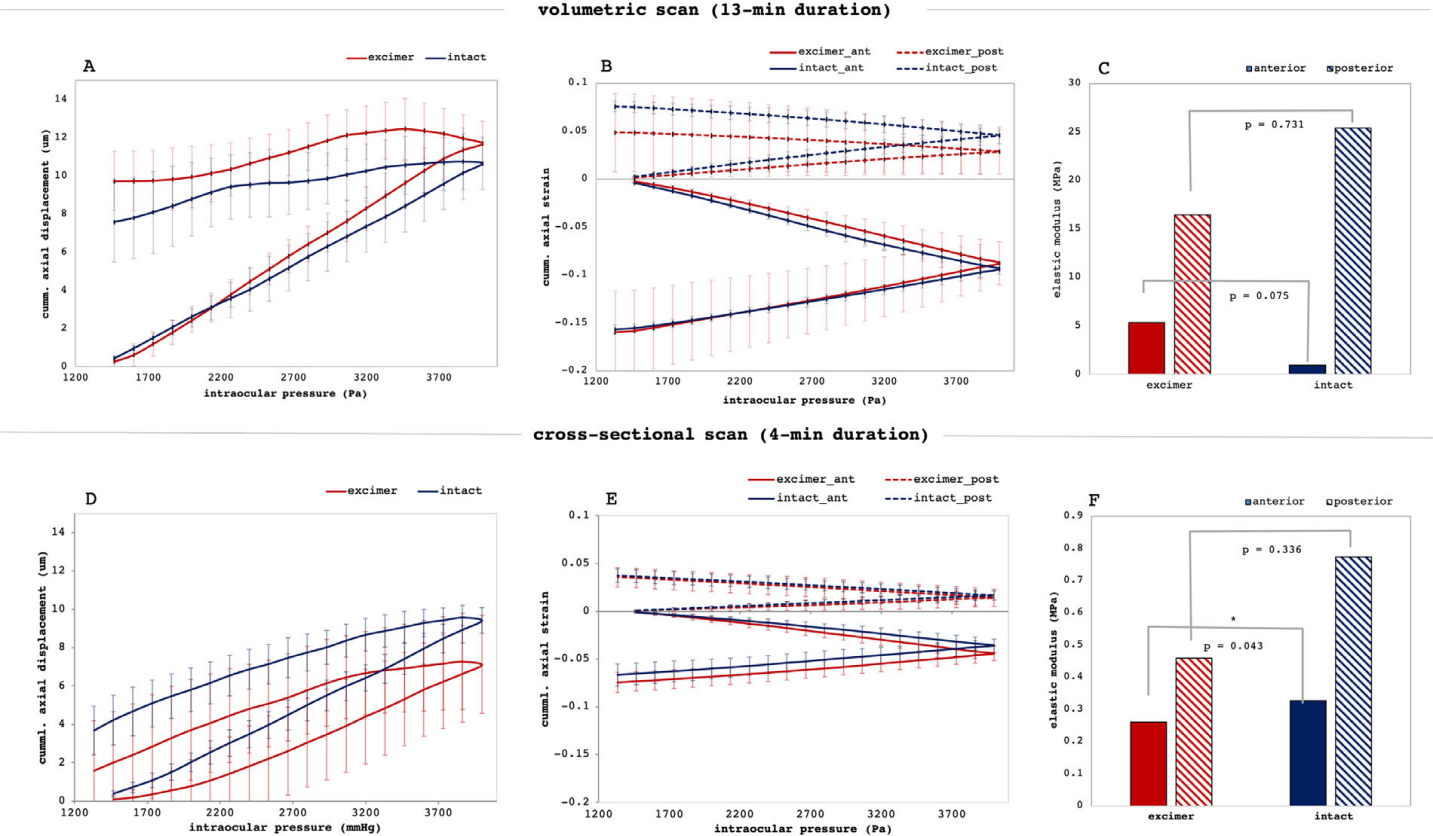
Biomechanical interpretation. (**A,D**) Accumulated vertical axial displacement of the central cornea as a function of IOP. Corneas did not fully recover after unloading. (**B,E**) Accumulated stress-strain diagram in the anterior and posterior cornea as a function of IOP. (**C,F**) Corresponding elastic moduli of the anterior and posterior cornea (*p<0.05). *Error bars* are omitted for clarity. Corneal strain and deformation were significantly lower the faster the measurement was conducted corresponding to a higher elastic modulus.

Figure 5 presents the accumulated corneal deformation response and elastic moduli for the complete sample set. Corneal apex (Figs. 5A, 5D) experienced on average a vertical displacement of 585 nm/mm Hg (set 1) and of 486 nm/mm Hg with Bowman, 439 nm/mm Hg without (set 2) during IOP increase. The induced displacement did only partially recover and remained at an elevation of 8.64 ± 2.02 µm (set 1) and 2.63 ± 1.95 µm (set 2) after IOP decrease. No significant differences were observed between corneas with and without Bowman's layer. The stress-strain diagram (Figs. 5B, 5E) presents the accumulated strain as a function of applied pressure both for the anterior and posterior stroma. A remarkably close to linear relation was observed, in which a steeper slope indicates a softer material. The corresponding elastic moduli (Figs. 5C, 5F) indicate that the anterior cornea behaves less stiff than the posterior cornea. Anterior stiffness in the anterior 50% of corneal thickness in samples with Bowman's layer was significantly (*P* = 0.043, set 2) higher than in corneas without. Differences were also observed between the two measurement sets performed at different speeds. A slower loading (set 1) was associated with a larger displacement and higher strain values, which is in agreement with viscoelastic material properties. Accordingly, the material behaves stiffer with faster loading.

**Figure 6.**
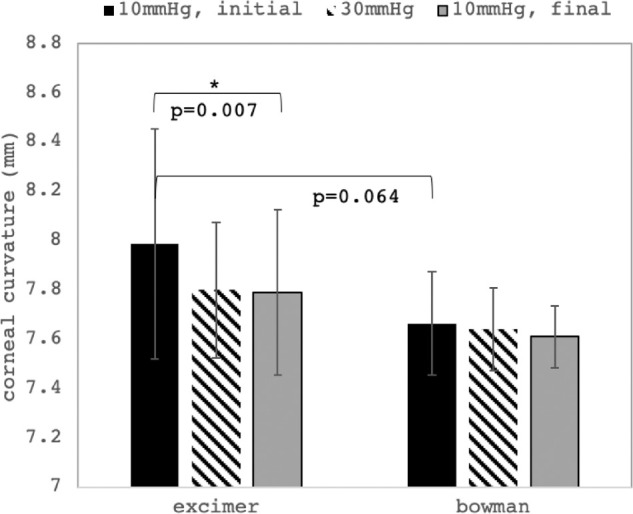
Corneal curvature changes. Mean corneal curvature at three distinct times: at 10 mm Hg before IOP modulation, at 30 mm Hg at maximal IOP, and at 10 mm Hg after IOP modulation. A significant reduction of corneal curvature after IOP modulation was observed in corneas without Bowman's layer (*p<0.05).

Figure 6 presents curvature values describing initial corneal shape at 10 mm Hg, changes induced by increasing IOP to 30 mm Hg, and remaining changes after IOP decreased back to 10 mm Hg. Samples without Bowman's layer showed a significant (*P* = 0.007) reduction of corneal curvature after the IOP modulation, whereas samples with Bowman's layer did not. A trend was observed that curvature of corneas with Bowman's layer were smaller than those without (7.66 ± 0.21 mm vs. 7.99 ± 0.47 mm, *P* = 0.064).

Overall, largest biomechanical differences were observed between the anterior and posterior corneal stroma, whereas the presence or absence of Bowman's layer could only account for subtle differences.

## Discussion

We were able to record spatially resolved corneal strain distribution over the entire stromal thickness in response to a physiological stress stimulus, with pressure loading amplitudes matching a clinically relevant IOP range (10–30 mm Hg). We demonstrated that strain maps could be retrieved from a single pressure change as little as 1 mm Hg. This high sensitivity makes the technique particularly interesting for future clinical applications. Detecting alterations in corneal strain distribution might be a useful diagnostic and screening parameter for corneal degenerative pathologies, as well as a follow-up tool to determine the progression of keratoconus and to quantify the effect of corneal cross-linking after treatment.

In the present study, by combining high-resolution OCE imaging with eye inflation, the impact of Bowman's layer could be studied not only in terms of strain distribution, and hence biomechanical stiffness, but also in terms of displacement and curvature changes. We found significant differences in axial strain amplitude, but also in strain distribution at specific locations in the anterior and posterior stroma between corneas with and without Bowman's layer. In contrast, no difference was observed in bulk displacement or radii of curvature changes, which is in agreement with previous literature that reported no effect of the presence or absence of Bowman's layer. Hence spatially resolved strain measurements by OCE can be considered a tool to reveal more subtle differences in corneal biomechanical functioning. The axial resolution of the displacement and strain maps had approximately the same dimension (16 and 26 µm, respectively) as Bowman's layer's thickness^35^ (17.7–20.0 µm). Thus the image resolution can be expected to be sufficient to identify localized effects of Bowman's layer. In some corneas, an anterior strain layer appeared (Fig. 1d) that resembled Bowman's layer. Nevertheless, as it occurred both in corneas with and without excimer ablation, we attribute this layer to result from dehydration effects during the measurement.

Even though differences in strain observed between corneas with and without Bowman's layer were located inside the stroma and not as expected on top of the anterior surface, we speculate that Bowman's layer might still contribute to stabilize the tissue, which is also evidenced by the fact that corneas with Bowman's layer removed presented a substantially higher standard deviation (e.g., Figs. 4F, 6). Also, at least in set 2 with a higher sample number, anterior strain amplitude in corneas with Bowman's layer was significantly smaller, indicating that peak strains are reduced. Interestingly, similar changes were found in the posterior corneal stroma.

The fact that corneal strain did not reverse its sign after IOP decrease is an unexpected finding. It implies that corneal strain could not recover, but instead proceeded to increase toward the same direction. Although viscoelastic properties can explain retarded deformation responses, it is uncommon that the deformation continues, whereas the load is already removed. We can rule out that the pressure chamber did not adequately transfer the current IOP, as displacement tracking clearly confirmed that the apex experienced an immediate downward displacement on IOP decrease. Theoretically, corneal strain could have been masked by an osmotic gradient, either (1) imposed by the fluid used to fill the anterior chamber, which was different from the cell culture medium the corneas were stored in; and/or (2) because the anterior surface was exposed to air during the measurement and no longer to the storage medium. Future studies in in vivo corneas are required to clarify these doubts. Still, the findings obtained in this study are relevant in terms of that most previously performed human corneal biomechanical characterization has been performed in ex vivo donor eyes, and thus likely experienced the same phenomenon.

The nearly linear stress-strain relationship observed in the current study is different from previous extensometry-based studies, which suggested a highly nonlinear stress-strain relation in corneal tissue. A major difference between these studies and ours is the applied stress amplitude, which was approximately 100 times lower in the current study (30 mm Hg ≈ 4 kPa), compared with previous extensometry tests^6^^,^^9^^,^^10^ (200–600 kPa). Thus current measurements were likely performed within the elastic region of the tissue, where Hook's law is applicable.

Surprisingly, the anterior cornea presented higher strain amplitudes than the posterior cornea. This is in contrast to previous literature that attributed the anterior stroma to a higher elastic modulus^36^ than the posterior stroma, but also to our previous study in freshly enucleated porcine eyes,^3^ in which the largest strain amplitudes were found in the posterior cornea. Although the reason for this might be ascribed to structural differences between human and porcine corneal tissue, it might also be a result of a substantially higher hydration level of human corneas in the current study. A limitation of this study is that corneal tissue did not have epithelial and endothelial cell layers increasing its susceptibility to hydration. Central corneas were on average 842 ± 90.5 µm thick, and hence had approximately a 60% larger volume than normally hydrated corneas. In addition, donors had different ages and various time had passed between Descemet's membrane stripping and the OCT measurement. Nonetheless, the same strain distribution profile—negative in the anterior and positive in the posterior cornea—was found in freshly enucleated porcine eyes, confirming the validity of using corneal tissue with epithelial and endothelial layers removed for the purpose of this study. Also, the boundary condition (i.e., fixed limbus in the anterior chamber in human corneas vs. whole eye globe inflation in porcine eyes) did not notably affect the strain distribution in the corneal periphery. In contrast, and as expected, corneal displacement measurements had a larger intersample variance due to their strong geometric dependency, for example, on corneal thickness, curvature, and mounting symmetry. This highlights the advantage of directly measuring tissue strain instead of geometric deformation parameters.

We observed a strong dependency of corneal strain and displacement amplitude on the speed the measurement was conducted at. In line with a viscoelastic material, at faster loading speeds the results indicated smaller strain and displacements corresponding to a higher stiffness. This points out the importance of standardizing loading velocity in future measurements.

## Conclusions

Strain maps as obtained from the presented OCE method are valuable inputs for ophthalmic modeling and simulation approaches. The observed linear stress-strain relationship between 10 and 30 mm Hg also suggests that a linear material model might be sufficient for refractive surgery simulations. Moreover, even if not directly at the location of Bowman's layer in the cornea, the presence or absence of Bowman's layer seems to induce a minor biomechanical effect when measured by quasi-static OCE. Most importantly*,* the strain distribution we observed in corneal tissue might have direct implications on corneal and refractive laser surgeries, as it will contribute to a better understanding on which layer might be best suited for tissue implantation or removal, as well as to a better distinction of healthy from pathological corneal tissue.

## Supplementary Material

Supplement 1

Supplement 2

## Supplementary Material

**Supplementary Video S1**. Time-lapse of morphological deformation. Intact corneal tissue undergoing IOP modulation from 10 to 30 mmHg in steps of 1 mmHg, and return. Note the particular behavior of the mid-stroma.
