# Supplementary material for: Quasi-Static Optical Coherence Elastography to Characterize Human Corneal Biomechanical Properties
Source: Invest Ophthalmol Vis Sci. 2020 Jun 15;61(6):29. doi: 10.1167/iovs.61.6.29 (PMC7415307; doi:10.1167/iovs.61.6.29)
Supplement: Supplement 1 [file iovs-61-6-29_s001.docx]

**
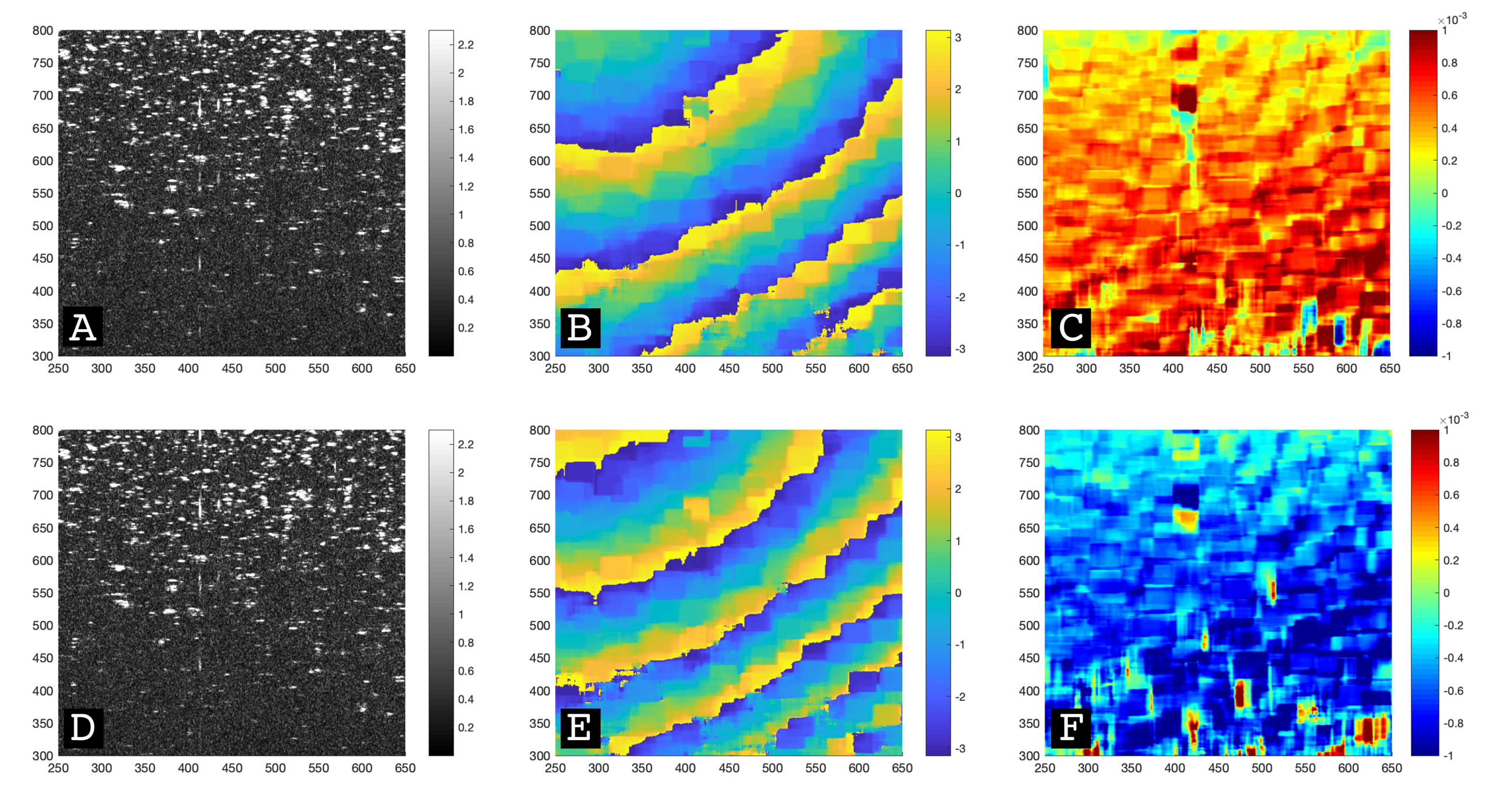
**

**Supplementary Figure 1. Validation of phase measurements in an isotropic material.** (**A-C**) Under lateral compression and corresponding vertical tension, the PDMS sample shows a phase change from negative towards positive in the direction bottom to top, corresponding to positive axial strain. (**D-F**) Under lateral tension and corresponding vertical compression, the PDMS sample shows an inverse phase change in the same direction, corresponding to negative axial strain.
